# RT-DETR-EVD: An Emergency Vehicle Detection Method Based on Improved RT-DETR

**DOI:** 10.3390/s25113327

**Published:** 2025-05-26

**Authors:** Jun Hu, Jiahao Zheng, Wenwei Wan, Yongqi Zhou, Zhikai Huang

**Affiliations:** School of Mechatronics & Vehicle Engineering, East China Jiaotong University, Nanchang 330013, China; zjh954thur@163.com (J.Z.);

**Keywords:** RT-DETR, emergency vehicle detection, CSPDarknet, MogaBlock, dynamic position bias, lightweight

## Abstract

With the rapid acceleration of urbanization and the increasing volume of road traffic, emergency vehicles frequently encounter congestion when performing urgent tasks. Failure to yield in a timely manner can result in the loss of critical rescue time. Therefore, this study aims to develop a lightweight and high-precision RT-DETR-EVD emergency vehicle detection model to enhance urban emergency response capabilities. The proposed model replaces ResNet with a lightweight CSPDarknet backbone and integrates an innovative hybrid C2f-MogaBlock architecture. A multi-order gated aggregation mechanism is introduced to dynamically fuse multi-scale features, improving spatial-channel feature representation while reducing the number of parameters. Additionally, an Attention-based Intra-scale Feature Interaction Dynamic Position Bias (AIDPB) module is designed, replacing fixed positional encoding with learnable dynamic position bias (DPB), improving feature discrimination in complex scenarios. The experimental results demonstrate that the improved RT-DETR-EVD model achieves superior performance in emergency vehicle detection under the same training conditions. Specifically, compared to the baseline RT-DETR-r18 model, RT-DETR-EVD reduces parameter count to 14.5 M (a 27.1% reduction), lowers floating-point operations (FLOPs) to 49.5 G (a 13.2% reduction), and improves precision by 0.5%. Additionally, recall and mean average precision (mAP50%) increase by 0.6%, reaching an mAP50% of 88.3%. The proposed RT-DETR-EVD model achieves a breakthrough balance between accuracy, efficiency, and scene adaptability. Its unique lightweight design enhances detection accuracy while significantly reducing model size and accelerating inference. This model provides an efficient and reliable solution for smart city emergency response systems, demonstrating strong deployment potential in real-world engineering applications.

## 1. Introduction

In modern smart city development and traffic management systems, the timely detection and rapid passage of emergency vehicles have become critical challenges that urgently require solutions. With the accelerating global urbanization and the continuous rise in road traffic volume, emergency vehicles (e.g., ambulances, fire trucks, and police cars) frequently encounter congestion when responding to emergencies. Failure to yield to these vehicles in a timely manner may result in the loss of golden rescue time. Therefore, establishing an efficient and reliable emergency vehicle detection system is not only essential for ensuring timely emergency response but also directly impacts urban public safety and the protection of lives and property. Traditional emergency vehicle detection methods primarily rely on manual visual recognition, acoustic signal-based detection, or sensor-based approaches such as RFID, infrared sensors, and Doppler radar. While these methods can achieve basic detection functionality under ideal conditions, their accuracy and real-time performance often fall short in complex real-world scenarios, such as adverse weather conditions, lighting variations, and occlusions. Moreover, conventional approaches typically demand high computational resources, making them difficult to deploy efficiently on edge devices for real-time processing.

To overcome the limitations of traditional detection methods, recent advancements in computer vision, image processing, and machine learning have led to increasing attention to deep learning-based detection approaches [1]. One of the earliest models applied to object recognition was R-CNN [2], which replaced the handcrafted feature extraction process in traditional object detection algorithms with feature extraction using convolutional neural networks (CNNs), significantly improving detection accuracy. Subsequently, Girshick et al. proposed Fast R-CNN [3] and Faster R-CNN [4], introducing various optimizations to enhance the efficiency of R-CNN. Further optimization algorithms, including You Only Look Once (YOLO) [5] and the Single Shot Multibox Detector (SSD) [6], have been integrated into the field of vehicle detection. While these algorithms have demonstrated outstanding performance in vehicle detection tasks, they still suffer from limitations, such as constrained feature extraction capabilities and difficulties in detecting small distant objects.

Therefore, beyond the academic contribution of enhancing detection accuracy and reducing model complexity, this study is motivated by real-world engineering challenges. Specifically, in many urban areas, emergency vehicles often experience significant delays due to insufficient real-time detection and prioritization at intersections. Existing roadside units (RSUs) frequently lack the computational resources to run heavy deep learning models. Hence, our goal is to develop a lightweight and high-precision emergency vehicle detection model that can be effectively deployed on low-power edge devices within intelligent transportation systems (ITSs), contributing to faster emergency response and reduced rescue delays.

Based on the aforementioned research progress, this study proposes an improved RT-DETR model for emergency vehicle detection. By adopting RT-DETR as the baseline model and introducing targeted enhancements, the objective is to develop a detection system that not only ensures high detection accuracy but also achieves superior real-time performance and adaptability to diverse environments. This system is designed to provide technical support for enhancing urban emergency response capabilities, demonstrating high feasibility. The main contributions of this study are as follows.

A CSPDarknet-based Backbone with an Innovative CSP-MogaBlock Hybrid Architecture: This study introduces CSPDarknet [7] as the new backbone network and proposes an innovative CSP-MogaBlock hybrid architecture. This design enhances the model’s ability to capture spatial and channel-wise feature interactions while simultaneously reducing overall model complexity.

AIDPB (Attention-based Intra-scale Feature Interaction Dynamic Position Bias) Module with Dynamic Position Bias for Adaptive Spatial Modeling: This study incorporates dynamic position bias (DPB) [8] and designs the AIDPB module to replace the AIFI module. By introducing learnable position-dependent attention bias, the proposed approach enables adaptive spatial modeling and more effective feature fusion across multiple scales. This design addresses the long-sequence extrapolation issues of traditional positional encoding, enhancing feature discrimination in occlusion and deformation scenarios.

The rest of the paper is organized as follows. Section 2 reviews related work on vehicle detection, with a special focus on emergency vehicle detection. Section 3 details the methodology and architecture of the improved RT-DETR-EVD model. Section 4 describes the experimental setup and evaluation metrics that comprehensively assess the effectiveness of the improved model. Section 5 discusses future research directions and provides insights into the ongoing challenges and future research trends in the field. Finally, Section 6 summarizes the results of this study.

## 2. Related Work

### 2.1. Deep Learning-Based Generic Vehicle Detection

In recent years, extensive research has been conducted by scholars worldwide to enhance the accuracy of general vehicle detection while improving model efficiency. Bie et al. [9] integrated the Squeeze-and-Excitation (SE) attention mechanism [10] into the backbone network of YOLOv5n-L to mitigate environmental noise interference. Kang et al. [11] proposed a type-1 fuzzy attention (T1FA), in which fuzzy entropy is introduced to re-weight the feature map in order to reduce the uncertainty of the feature map and facilitate the detector’s focus on the target center as a way to effectively improve the accuracy of vehicle detection. Zhang et al. [12] proposed an improved YOLOv5 approach for vehicle detection in diverse traffic scenarios. By utilizing the Flip–Mosaic algorithm, they enhanced small object detection and reduced occlusion-related misdetection rates. Cai et al. [13] introduced YOLOv4-5D, replacing the final layer of CSPDarknet53 with deformable convolution to improve detection accuracy. Li et al. [14] enhanced the YOLO model with a multi-scale dehazing module (MSRCR), ensuring high detection accuracy and real-time performance even in foggy conditions. Dong et al. [15] combined the Convolutional Block Attention Module (CBAM) with the CIOU Loss bounding box regression function to improve target localization precision and detection accuracy. Pan et al. [16] introduced LVD-YOLO, a lightweight vehicle detection model leveraging EfficientNetv2 as its backbone architecture. The model simultaneously reduces parameter count while enhancing feature extraction capabilities. Yan et al. [17] optimized the CSP module by leveraging dense connections and channel attention mechanisms to mitigate the vanishing gradient problem, enhance feature propagation, and reduce model parameters. Finally, Wang et al. [18] proposed an enhanced YOLOv4 model employing an anchor-free mechanism to optimize anchor box matching. This approach improves adaptability to target size variations and enhances real-time detection performance in challenging weather conditions, particularly for multi-scale object recognition.

Furthermore, in response to practical application scenarios with limited data availability, several studies have incorporated lightweight few-shot learning strategies. Pan [19] introduced global average pooling (GAP) and one-dimensional convolution techniques, which significantly reduced model storage requirements and training time while sacrificing minimal detection accuracy. Beyond few-shot recognition approaches, Liu [20] developed an efficient, low-consumption GAN framework for pavement distress recognition, achieving up to 94.8% reduction in model parameters while maintaining a high classification accuracy of 85.4%. These methodologies provide valuable reference for enhancing the detection of scarce-category objects (such as emergency vehicles) and contribute to deployment practices under resource-constrained conditions.

In addition to model-level improvements, recent work has emphasized the role of dataset diversity in enhancing detection robustness. RSUD20K [21] is a large-scale dataset collected from Bangladeshi roads, featuring over 20,000 high-resolution images and 130,000 bounding boxes across 13 object categories. It captures dense scenes, narrow streets, and varied weather, offering a challenging benchmark for evaluating object detectors in complex and geographically distinct environments. Although it does not focus specifically on emergency vehicles, it provides useful insights for developing detection models that generalize across diverse real-world conditions.

### 2.2. Dedicated Emergency Vehicle Inspection System

However, research on emergency vehicle detection based on deep learning remains relatively limited. Baghel [22] proposed a two-stage V-EVD system, where a detection module first identifies vehicles, followed by a classification module for vehicle recognition. After testing on a small custom dataset, the system achieved an accuracy of 91.6%. Similarly, Roy and M. S. Rahman [23] introduced a two-stage approach, while Goel [24] reviewed the performance of various object detection algorithms and highlighted YOLO as a promising candidate for V-EVD tasks due to its exceptional processing speed. Notably, beyond vehicle recognition, intelligent identification of traffic bottlenecks is equally crucial in urban emergency response systems. Pan [25] proposed a deep learning-based street view image analysis method, developing a classification model built on Vision Transformer architecture to enable automatic identification of ambulance passageways in urban streets. The experimental results demonstrated that the four-headed single-sequence encoder model achieved an accuracy of 75.65% on the original dataset, with the segmentation rate improved to 77.42%, exhibiting substantial deployment potential in practical applications across Beijing urban districts. This work extends the research boundaries of emergency vehicle detection and illustrates the comprehensive value of deep learning models in urban emergency traffic management.

### 2.3. Transformer-Based Target Detection

The introduction of the Transformer architecture has provided new insights into addressing the aforementioned challenges. The Transformer-based object detection model DETR [26] combines the local feature extraction capability of CNNs with the global modeling ability of Transformers, effectively handling object detection tasks and pioneering a new paradigm in the field. This method adopts an encoder–decoder architecture and introduces a bipartite matching strategy, enabling direct prediction of object locations in an image without the need for complex processes such as proposal generation and non-maximum suppression (NMS) in traditional object detection pipelines. However, DETR suffers from several limitations due to its reliance on the large-scale Transformer architecture, including low real-time detection performance, slow training convergence, and suboptimal detection accuracy. To address these issues, RT-DETR [27] incorporates fine-grained feature extraction, deformable attention mechanisms, and other optimization strategies, achieving fully end-to-end object detection. These improvements enable RT-DETR to attain higher training accuracy with fewer iterations, effectively mitigating DETR’s slow convergence problem. Compared to the YOLO model, RT-DETR not only achieves breakthroughs in detection accuracy but also significantly reduces computational complexity. Its outstanding performance across multiple benchmark datasets has encouraged researchers to explore its applications in real-world scenarios, offering a novel and feasible solution for various domains. However, despite RT-DETR’s significant advancements in real-time performance and training efficiency, it still faces challenges in analyzing complex scenes, such as detecting small objects, handling severe occlusions, and mitigating motion blur. Furthermore, its capability to fuse multi-level features still has room for optimization. These challenges indicate that while RT-DETR demonstrates excellent real-time detection capabilities, improvements in feature processing and fusion strategies are necessary to better adapt to complex and dynamic detection tasks.

## 3. Materials and Methods

### 3.1. Data Acquisition

To evaluate the effectiveness and applicability of our method for emergency vehicle detection, this study utilizes the large-scale and diverse Recognition of Government Vehicles in Russia dataset, provided by Kaggle (k1rsn7, 2023). This dataset exhibits significant diversity, encompassing vehicle images captured under various weather conditions (sunny, cloudy, rainy), and multiple camera angles. Furthermore, it includes a wide range of complex scenarios, such as traffic congestion, partial occlusion, and motion blur, enhancing the model’s adaptability to real-world applications.

**Figure 1:**
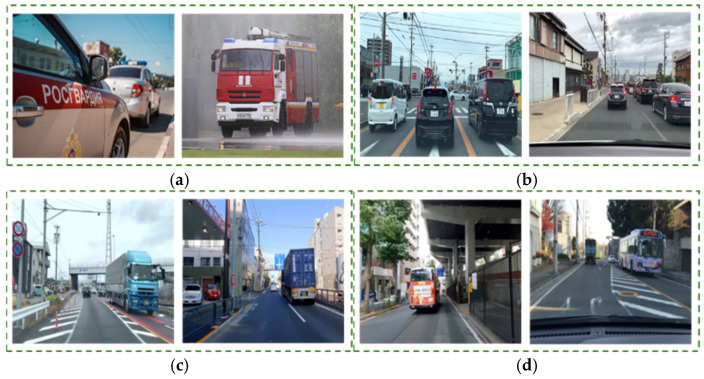
Overview of the dataset. (**a**) Government Car. (**b**). Car. (**c**). Truck. (**d**) Bus.

The dataset consists of 6240 vehicle images with varying resolutions and defines four categories of detection targets: government vehicles, regular vehicles, trucks, and buses. Among them, government vehicles—comprising fire trucks, police cars, and ambulances—are the focus of this study as emergency vehicles. Sample images from the dataset are illustrated in Figure 1.

The dataset is randomly divided into three subsets at an 8:1:1 ratio: 4860 images for training, 681 images for validation, and 699 images for testing. The annotations follow the YOLO format. The detailed data distribution is presented in Table 1.

After analyzing the original dataset, it was observed that the bus category had relatively few samples in the validation set, which led to a decline in overall detection accuracy for the emergency vehicle detection task. To mitigate this issue, we adjusted the dataset partitioning strategy by merging the validation and test sets, resulting in a larger validation set. This adjustment ensures a more balanced sample distribution across all categories, enhancing the model’s generalization ability and improving detection accuracy.

The adjusted dataset contains a total of 6240 images, with 4860 images allocated for training and the remaining 1380 images for validation. Compared to the original 8:1:1 random split, the new scheme adopts an 8:2 ratio, allowing the validation set to encompass a more diverse set of samples for a more accurate evaluation of the model’s performance. The revised data distribution is presented in Table 2. During training, to reduce memory consumption, all images were resized to a uniform resolution of 640 × 640 pixels, enhancing both training efficiency and model performance.

### 3.2. RT-DETR Baseline Network Architecture

**Figure 2:**
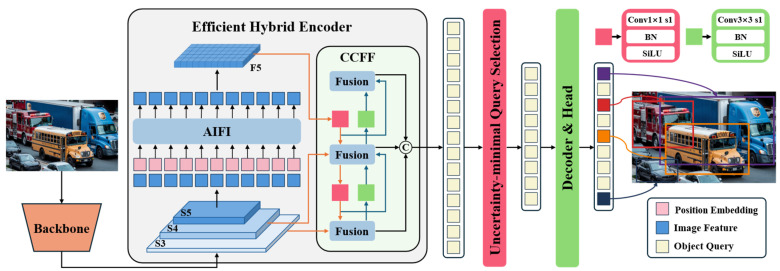
RT-DETR network architecture diagram.

As a next-generation object detection model based on the Transformer architecture, RT-DETR achieves an outstanding balance between real-time performance and detection accuracy. The detailed network structure of RT-DETR is illustrated in Figure 2. By introducing an innovative feature processing mechanism, RT-DETR adopts a hierarchical strategy for multi-scale feature handling: it first enhances features within each scale and then performs cross-scale feature integration. This approach not only significantly reduces computational overhead but also completely eliminates the post-processing steps found in traditional detectors, resulting in a smoother and more efficient inference process. From an architectural perspective, RT-DETR consists of three key modules: a feature extraction backbone, a hybrid feature enhancement encoder, and a Transformer decoder with an auxiliary prediction mechanism. For the backbone network, the model employs an efficient CNN architecture, which can be either the widely used ResNet [28] series or the specially optimized HGNet [29]. This choice is driven by the need for real-time performance, as CNNs demonstrate superior efficiency and practicality in the feature extraction stage compared to other architectures. In the feature processing stage, RT-DETR incorporates two innovative modules: the Attention-Enhanced Intra-Scale Feature Interaction (AIFI) Module and the Convolution-Driven Cross-Scale Feature Fusion (CCFM) Module. The AIFI module vectorizes deep features using a lightweight Transformer encoder, followed by feature reconstruction through a feedforward network. Finally, the CCFM module intelligently fuses multi-level features to generate a rich feature representation. For the detection head, RT-DETR adopts the denoising strategy inspired by DINO [30], which significantly improves sample matching quality and accelerates the model’s convergence.

From a functional perspective, the three core components of RT-DETR operate independently while maintaining close collaboration. The backbone and feature processing modules serve as the foundation of the model, directly influencing its final detection performance. The backbone network is responsible for extracting multi-level visual features from input images, while the feature processing module plays a crucial role in feature optimization and fusion. Although the original RT-DETR employs a CNN-based backbone with strong feature extraction capabilities, it still faces practical challenges, such as a large number of parameters and high computational costs. Additionally, the attention mechanism in the AIFI module exhibits computational redundancy across different feature levels, increasing the complexity of model training to some extent. It is worth noting that while the CCFM module shares structural similarities with the Path Aggregation Feature Pyramid Network (PaFPN) [31], it presents unique challenges in feature processing. In object detection, high-quality features must simultaneously preserve fine-grained details and rich semantic information. Traditional feature pyramid structures often suffer from semantic degradation when processing high-level features, whereas the bottom-up feature fusion process may lead to detail loss in low-level features, posing significant challenges for small object detection. Therefore, future research will focus on enhancing both the backbone feature extraction network and the neck network to address these issues, ultimately improving the accurate recognition of emergency vehicles.

### 3.3. RT-DETR-EVD Improved Model Architecture

**Figure 3:**
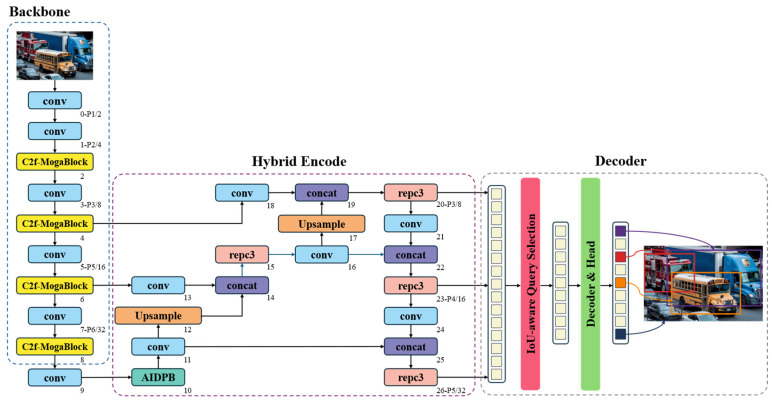
RT-DETR-EVD network architecture diagram.

To address the issues of large parameter size, high computational cost, and suboptimal detection performance in the RT-DETR model, this study proposes the RT-DETR-EVD model. The detailed architecture of the improved model is illustrated in Figure 3.

First, to address the two major challenges of the ResNet-18 backbone in feature extraction—namely, its large number of parameters and its limited capability in global contextual feature extraction—this study adopts CSPDarknet as the new backbone network and further proposes an innovative C2f-MogaBlock hybrid architecture. The C2f-MogaBlock leverages a multi-branch adaptive feature aggregation mechanism, enabling the intelligent capture and integration of multi-scale feature information. Additionally, its residual structure ensures effective information transmission, thereby enhancing feature representation while maintaining low computational complexity. Moreover, the original AIFI module employs fixed positional encoding during feature fusion, which restricts its ability to adapt to dynamic spatial relationships. To overcome this limitation, this study introduces a novel AIDPB module, which enhances the AIFI module by incorporating dynamic position bias (DPB). By integrating learnable position-dependent attention bias, the AIDPB module enables adaptive spatial modeling, allowing the attention mechanism to dynamically adjust its importance based on the relative spatial relationships of different positions. This results in more effective feature integration across multiple scales, effectively mitigating potential feature loss issues in complex traffic scenarios. In summary, the improved RT-DETR-EVD model significantly enhances both global and local feature understanding while achieving lightweight and efficient model design, making it better suited for real-time emergency vehicle detection.

#### 3.3.1. Backbone Network: CSP-MogaBlock Feature Extraction Architecture

**Figure 4:**
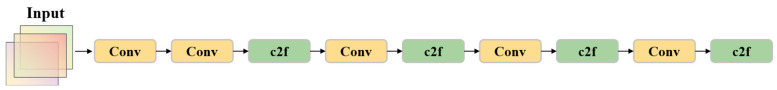
CSPDarknet backbone network diagram.

In the field of object detection, model lightweighting and deployment efficiency have always been key areas of research. The traditional RT-DETR model adopts ResNet or HGNet as its feature extraction backbone, which, while demonstrating strong feature representation capabilities, still suffers from high computational overhead due to its complex network hierarchy and large number of parameters. This inefficiency is particularly evident in multi-scale feature fusion, making it difficult to meet the practical demands of resource-constrained environments, such as deployment on mobile devices. To address this challenge, we introduce CSPDarknet from YOLOv8 [32] as the new backbone network. The core innovation of this improvement lies in its Cross-Stage Partial (CSP) network architecture, which redesigns the gradient flow path to significantly reduce computational costs while enhancing the spatial and channel-wise feature interactions. The optimized feature transmission mechanism in CSPDarknet enables the model to more accurately capture critical features of emergency vehicles while maintaining low computational complexity and enhanced feature representation capabilities. The structure of the CSPDarknet backbone network is illustrated in Figure 4.

**Figure 5:**
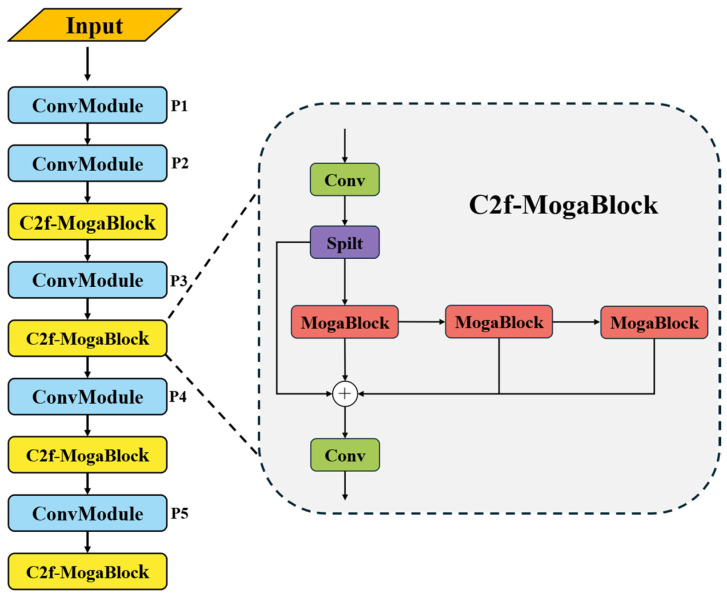
Improved backbone network architecture diagram.

To further enhance model performance, we introduce a deep structural modification to the C2f module in CSPDarknet. While the original C2f architecture leverages multiple residual connections to facilitate gradient information flow, its core component—the traditional bottleneck structure—often struggles to fully capture fine-grained features in complex scenarios, such as emergency vehicle detection. Based on this observation, we propose an innovative C2f-MogaBlock hybrid architecture. This design seamlessly integrates the MogaBlock module—known for its exceptional feature aggregation capability in MogaNet [33]—with the bottleneck framework of C2f, creating a more powerful feature extraction unit. The multi-branch adaptive feature aggregation mechanism in MogaBlock enables the intelligent capture of multi-scale features, while the retained residual structure ensures efficient information transmission. This improvement not only enhances the network’s adaptability to complex environments but also improves feature extraction efficiency, enabling the model to better handle various challenging scenarios in emergency vehicle detection. The detailed architecture of this hybrid design and the modified backbone network framework are illustrated in Figure 5.

**Figure 6:**
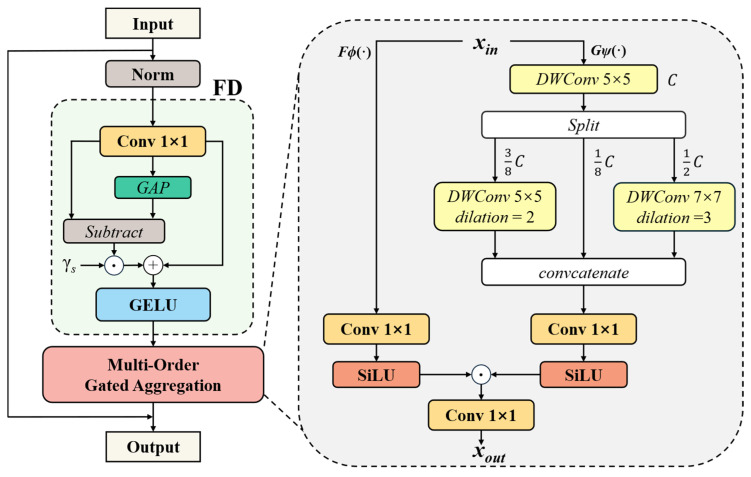
MogaBlock architecture diagram.

We propose MogaBlock, a sophisticated two-stage cascaded architecture designed to enhance feature representation learning through feature decomposition and multi-order gated aggregation. As illustrated in Figure 6, the module first employs convolutions with varying dilation rates to simultaneously extract local fine-grained details and global semantic features. Subsequently, an adaptive gating mechanism dynamically learns and fuses these multi-scale features, significantly improving the network’s multi-scale perception capabilities as follows:

(1) $$Z=X+Moga\left(FD\left(Norm\left(X\right)\right)\right)$$

where ***X*** is the input feature map and Norm(***X***) denotes the normalization of the input ***X***. FD represents the feature decomposition module, while Moga denotes a multi-order gated aggregation module comprising the gating *Fϕ*(·) and context branch *Gψ*(·).

The feature decomposition (FD) module employs a two-stage strategy to effectively extract multi-scale representations. Initially, a 1 × 1 convolutional layer transforms the input feature ***X*** into an intermediate representation ***Y***, capturing local feature nuances as described in Equation (2). Subsequently, global average pooling (GAP) is applied to ***Y***, distilling global statistical characteristics outlined in Equation (3). This architectural design enables concurrent preservation of fine-grained textural details and holistic semantic information.

(2) $$Y={Conv}_{1\times 1}\left(X\right)$$

(3) $$Z=GELU\left(Y+\gamma_{s}⊙\left(Y-GAP\left(Y\right)\right)\right)$$

where ***X*** is the input feature and ***Y*** is the new feature after processing by a convolution operation. $\gamma_{s}$∈*R*^*C*×1^ represents a learnable parameter vector initialized to zero. By reweighting the complementary interaction components ***Y*** − *GAP*(***Y***), the FD module further enhances spatial feature diversity.

The multi-order gated aggregation (Moga) module introduces an innovative dual-branch architecture. The aggregation branch dynamically generates adaptive weights, while the contextual branch leverages multi-scale depth-wise convolutions to extract comprehensive feature representations. Both branches employ the Sigmoid Linear Unit (SiLU) activation, strategically balancing gating mechanisms with numerical stability during model training.

In the implementation, the contextual branch performs hierarchical feature processing on the input tensor Xin. Initially, a 5 × 5 depthwise convolution extracts foundational features. The resulting feature map is then strategically partitioned into three channel-wise segments with proportions of 1/8, 3/8, and 1/2. Each segment undergoes distinct depthwise convolution transformations: the first segment preserves core representational information, the second employs a 5 × 5 convolution with a dilation rate of 2, while the third utilizes a 7 × 7 convolution with a dilation rate of 3. Subsequently, these feature representations are reassembled along the channel dimension and refined through the aggregation branch’s 1 × 1 convolution, ultimately generating the module’s output features as described in Equation (4). This sophisticated multi-scale feature extraction and adaptive aggregation mechanism substantially augments the model’s capacity to discern intricate spatial–contextual representations.

(4) $$Z=SilU\left(X\right)⊙SilU\left(Y\right)$$

where ***X*** represents the output of the aggregation branch, ***Y*** denotes the output of the context branch, and ***Z*** corresponds to the output of the feature aggregation module.

By adopting different dilation rates, the convolutional layers expand their receptive field without increasing computational complexity. This design enables the model to effectively capture contextual information across multiple scales, allowing for a more comprehensive understanding of both global and local features, particularly in complex scenes. Leveraging the advantages of the C2f module, we replace the first convolutional layer in the bottleneck module with MogaBlock. This modification not only reduces model complexity but also adaptively integrates multi-level interactive features, thereby extracting more distinguishable features across different scales. As a result, the model’s representational capacity and detection accuracy are further enhanced.

#### 3.3.2. AIDPB: A Novel Mechanism for Multi-Scale Feature Alignment

The Attention-Based Intra-Scale Feature Interaction (AIFI) module is a key component of the efficient hybrid encoder in RT-DETR, playing a crucial role in multi-scale feature integration. While effective, its original design relies on fixed positional encoding generated by sine functions to inject spatial information into features. However, this approach limits the module’s ability to adapt to dynamic spatial relationships, particularly when processing objects with varying scales and complex spatial layouts.

To overcome this limitation, this study introduces the AIDPB module, which enhances AIFI using dynamic position bias (DPB). By incorporating learnable position-dependent attention bias, AIDPB enables adaptive spatial modeling, allowing the attention mechanism to dynamically adjust its importance based on relative spatial positions. This enhancement facilitates more effective feature integration across different scales, significantly improving the model’s adaptability to complex detection scenarios.

**Figure 7:**
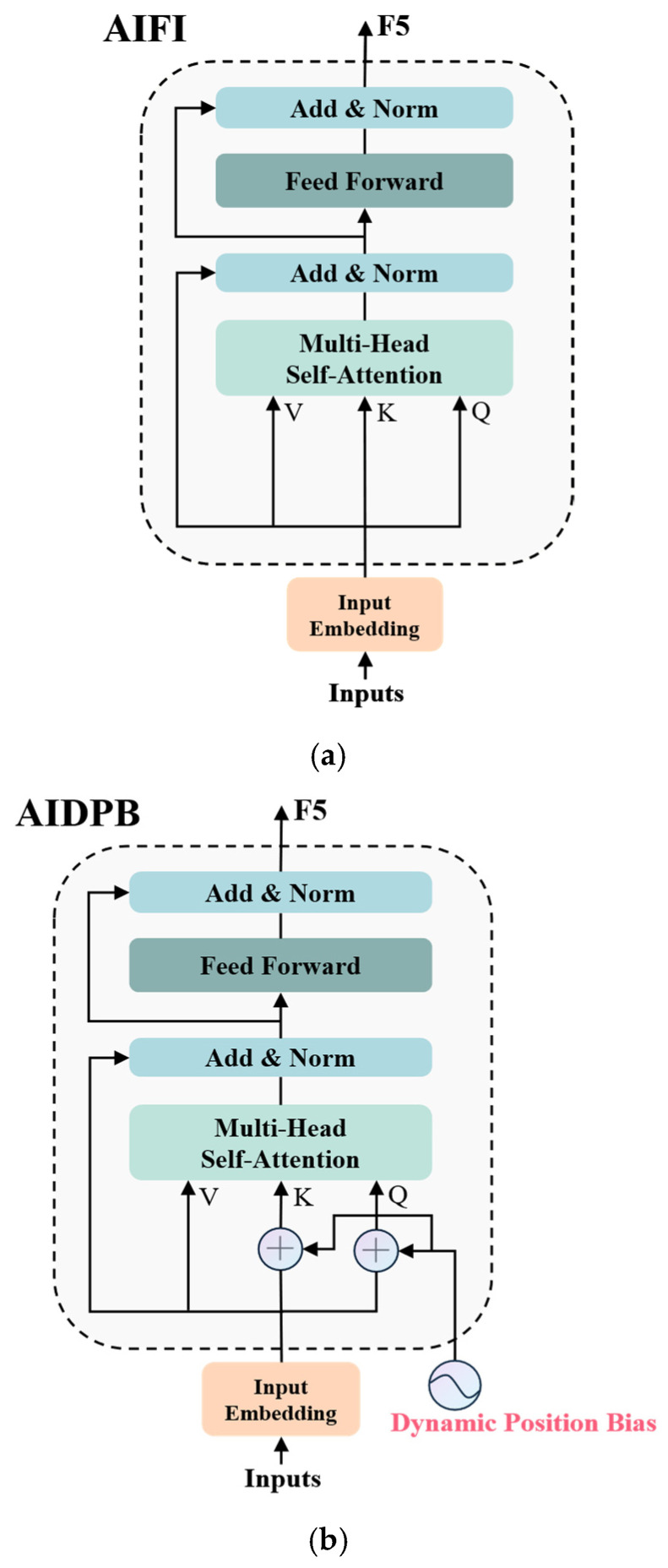
AIFI network architecture and AIDPB network architecture diagram. (**a**) Schematic diagram of the AIFI structure. (**b**) Schematic diagram of the AIDPB structure.

Dynamic position bias (DPB) is an innovative deep learning technique designed to overcome the limitations of traditional fixed positional encoding, particularly in handling variable-length sequences. In this study, we design the AIDPB module to achieve more adaptive position modeling, as illustrated in Figure 7. Unlike traditional fixed positional encodings, which use static representations, DPB dynamically learns positional relationships, allowing the model to adaptively adjust the association strength between different positions and better capture positional dependencies within a sequence.

**Figure 8:**
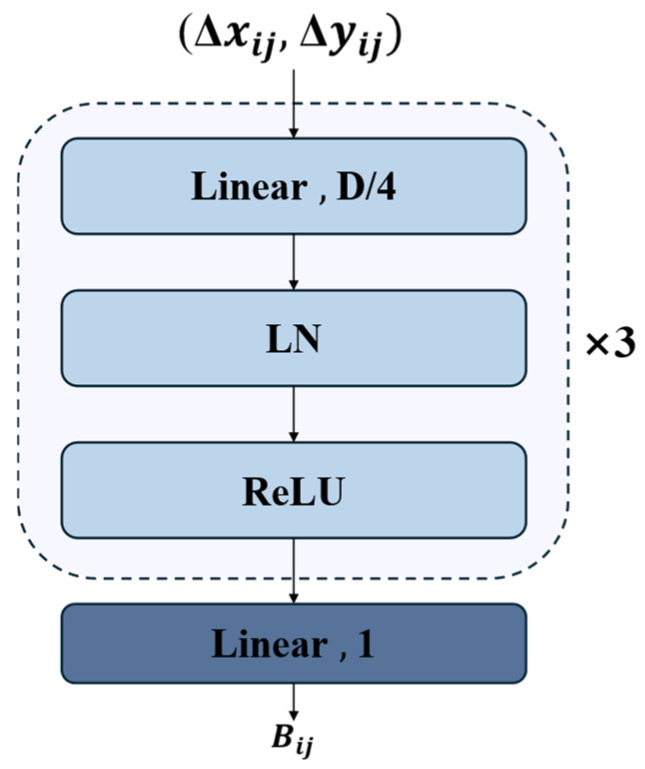
DPB architecture diagram.

As shown in Figure 8, the DPB structure takes the relative position offsets (**Δ*x_ij_***, **Δ*y_ij_***) as inputs and processes them through a carefully designed network architecture. Specifically, the inputs first pass through a linear layer with a dimension of D/4, followed by three repeated modules, each consisting of Layer Normalization (LN) and ReLU activation. Finally, a linear layer with an output dimension of 1 generates the position bias value ***B_ij_***. This architecture, with its compact design, dimensionality reduction (D/4), and simple network structure, effectively learns spatial relationships embedded in positional offsets while maintaining computational efficiency.

In the traditional Transformer architecture, the attention mechanism is typically computed following Equation (5).

(5) $$Attention=Softmax\left(\frac{QK^{T}}{\sqrt{d}}+B\right)V$$

where ***Q***, ***K***, and ***V*** represent the query, key, and value matrices, respectively. ***d*** denotes the model dimension, and ***B*** represents the bias term.

To enhance the attention mechanism in AIFI, we design the AIDPB module, an MLP-based module capable of dynamically generating relative position bias. Specifically, we modify the attention computation by replacing the bias term BB with the formulation given in Equation (6).

(6) $$B_{i,j}=DPB\left(\Delta x_{ij},\Delta y_{ij}\right)$$

where ***B_ij_*** represents the bias value between position ***i*** and position ***j***, while **Δ*x_ij_*** and **Δ*y_ij_*** denote the relative positional differences in the x-direction (horizontal) and y-direction (vertical), respectively.

The AIFI-DPB module, based on a scale-interaction mechanism with dynamic position bias (DPB), utilizes a lightweight MLP to adaptively generate spatial dependency weights, effectively addressing the long-sequence extrapolation bottleneck of traditional positional encoding in emergency vehicle detection tasks. Its dynamic learning mechanism accurately models the spatiotemporal relationships between vehicles (e.g., vehicle platooning and emergency lane changes), enhancing feature discrimination in challenging scenarios, such as occlusion and deformation. This approach significantly improves detection accuracy (mAP) and enhances the robustness of the model in complex environments.

## 4. Results and Analysis

### 4.1. Experimental Configuration

The experimental platform used in this study is Ubuntu 20.04, with all experiments conducted based on the PyTorch 10.0 deep learning framework using Python as the programming language. The primary hardware configuration of the computing system is as follows: CPU, 12 vCPU Intel^®^ Xeon^®^ Silver 4214R @ 2.40 GHz, operating system, Windows 11 (64-bit), and GPU, NVIDIA GeForce RTX 3080 Ti (12 GB).

Regarding the training strategy, the input image size is set to 640 × 640. The batch size is configured as 4, and the learning rate follows a cosine annealing decay schedule. The model is trained for a total of 200 epochs on the training dataset without freezing any layers. The initial learning rate is set to 0.0001, the momentum is configured as 0.9, and the weight decay rate is 0.0001. The main hyperparameter settings are detailed in Table 3.

### 4.2. Model Evaluation Metrics

Considering the characteristics of object detection tasks, the evaluation metrics used in this study include precision (*P*), recall (*R*), average precision (*AP*), and mean average precision (*mAP*) [34,35]. The computation methods for these metrics are as follows::

(7) $$Precision=\;\frac{TP}{TP+FP}$$

(8) $$Recall=\;\frac{TP}{TP+FN}$$

(9) $$AP=\;\int_{0}^{recall}Precision$$

(10) $$mAP=\;\frac{1}{n}\sum_{i=1}^{n}AP_{i\;}$$

In the evaluation metrics, precision (***P***) quantifies the ratio of correctly detected objects to the total predicted objects, while recall (***R***) measures the proportion of correctly identified ground truth objects. Key performance indicators include true positives (***TP***), false positives (***FP***), and false negatives (***FN***). Average precision (***AP***) represents the area under the precision–recall (***PR***) curve, computed by interpolating precision values across multiple confidence thresholds. ***AP*** serves as a comprehensive metric for assessing model performance across various recall levels, with values approaching 1 indicating superior detection capabilities. Mean average precision (***mAP***) extends this evaluation by averaging ***AP*** across all object categories, providing a holistic assessment of multi-class detection performance. To comprehensively validate model efficacy, additional metrics are employed, including model parameters, inference speed (frames per second, ***FPS***), and computational complexity (***FLOPs***), enabling a multidimensional performance evaluation.

### 4.3. Comparison of Backbone Networks in Baseline Models

As a core component of deep detection models, the design of the backbone network directly affects the model’s representation capability and task performance. Considering that RT-DETR commonly employs the ResNet series or Baidu’s self-developed HGNet as backbone networks, this study aims to identify a relatively balanced backbone in terms of accuracy and model complexity. Based on the emergency vehicle dataset, we select the ResNet series (including four depth variants: 18, 34, 50, and 101) and the lightweight architecture HGNetV2 as baseline comparisons. The quantitative analysis focuses on the following dimensions: (1) model parameters (Params), representing storage requirements; (2) floating-point operations (FLOPs), reflecting computational efficiency; and (3) mean average precision (mAP), measuring detection performance. Table 4 presents the detailed module configurations of each architecture along with their quantitative evaluation results on the standard test set, revealing the trade-off between model complexity and detection accuracy.

The experimental results indicate that among the evaluated backbone networks—ResNet18, ResNet34, ResNet50, and the original RT-DETR backbone HGNetV2—ResNet18 demonstrates a significant advantage in parameter efficiency due to its relatively shallow network architecture. Specifically, ResNet18 has a total parameter count of approximately 19.9 M, which is substantially lower than ResNet34 (31.1 M) and ResNet50 (42.0 M). In terms of computational complexity, ResNet18 achieves the lowest floating-point operations (FLOPs) at just 57.0 GFLOPs, making it the most computationally efficient among all compared models. This characteristic makes ResNet18 particularly suitable for embedded systems and real-time detection tasks, where efficiency is critical. Although ResNet18’s mean average precision (mAP) is approximately 87.8%, which is only marginally lower than ResNet101 (87.9%), its compact model size and reduced computational overhead make it an optimal choice when balancing detection accuracy and computational efficiency. Therefore, to meet the high-performance requirements of real-time emergency vehicle detection, this study selects ResNet18 as the backbone feature extraction network of the baseline model and will use it as the foundation for further model optimization.

### 4.4. Visual Recognition of the Heatmap

**Figure 9:**
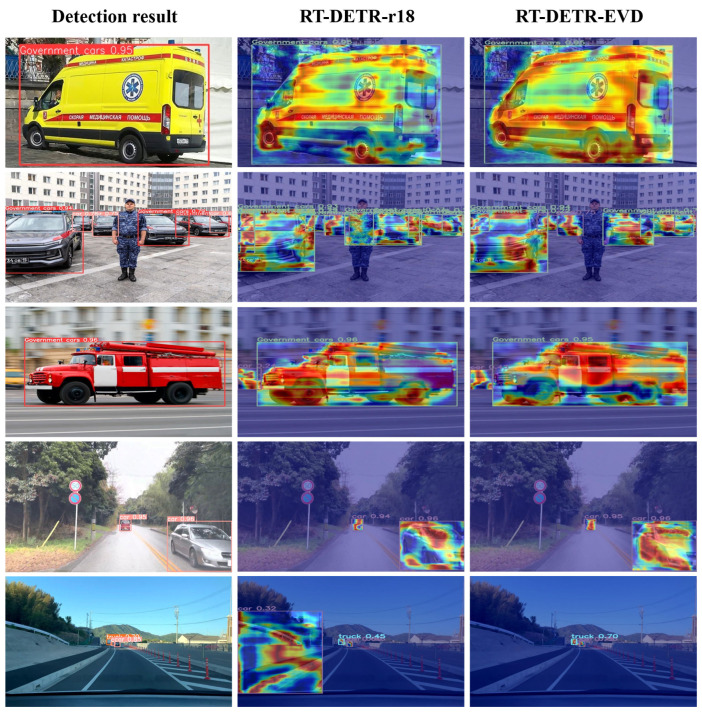
Detection results and Grad-CAM visualization.

With the widespread application of deep learning models across various fields, understanding and interpreting their detection capabilities has become a critical challenge. Enhancing model explainability not only helps researchers gain deeper insights into the model’s decision-making process but also improves trust in model predictions. To intuitively demonstrate the effectiveness of the proposed model in learning emergency vehicle features, this study employs the Grad-CAM [36] visualization technique to analyze the detection results of RT-DETR-r18 and RT-DETR-EVD, as shown in Figure 9. The core concept of Grad-CAM is to utilize the feature maps from the last convolutional layer of a deep convolutional neural network (CNN) and compute the gradient-based importance of different regions. By calculating gradient-weighted activations for a specific class, Grad-CAM identifies the most influential neurons that contribute to the model’s decision-making process. In the generated heatmaps, red regions indicate areas that contribute significantly to the prediction, whereas blue regions have a lower impact. This visualization helps in analyzing the model’s attention mechanism and understanding the basis of its decisions.

As shown in Figure 9, the heatmap visualization results clearly indicate that the original RT-DETR-r18 model exhibits scattered attention when detecting emergency vehicles, failing to effectively focus on the target. In contrast, the optimized RT-DETR-EVD model demonstrates a significant improvement in attention concentration, reducing the focus on irrelevant image regions. Notably, in challenging scenarios, such as overlapping emergency vehicles and pedestrians or vehicles positioned at the edge of the road, the original model is prone to misclassification. This issue is likely caused by the loss of low-level features during feature fusion, leading to insufficient semantic information. The proposed improved model exhibits remarkable advantages in feature extraction and learning. It is capable of more accurately capturing and extracting target features while effectively filtering out irrelevant contextual information, thereby mitigating misclassification issues. The heatmaps further illustrate that the optimized model successfully highlights emergency vehicles (marked in red) and maintains robust detection performance, even in complex traffic environments with multiple objects.

In the final test visualization results, the optimized model accurately localizes key regions in the heatmaps, demonstrating its ability to integrate rich semantic information while effectively understanding both global and local features. Even in complex traffic scenarios, the model successfully detects emergency vehicles with high accuracy, ensuring precise identification and reliable emergency vehicle recognition.

### 4.5. Ablation Experiments

To systematically evaluate the contribution of each innovative module to the model’s performance, we designed a series of progressive ablation experiments. Using RT-DETR-r18 as the baseline model, we conducted four sets of ablation experiments, gradually incorporating the proposed improvement modules, including MogaBlock, AIDPB, and others. The experimental results, presented in Table 5, clearly demonstrate the performance improvements contributed by each module, providing strong validation of the effectiveness of our proposed enhancements.

As shown in Table 5, Improvement 1 introduces CSPDarknet as the new backbone network architecture and integrates a novel CSP-MogaBlock hybrid structure. This design maintains a stable mean average precision (mAP) while successfully reducing the parameter count from 19.9 M in the baseline RT-DETR-r18 model to a more efficient 14.7 M while controlling the computational complexity at 49.7 G FLOPs. The CSP-MogaBlock architecture significantly enhances semantic feature richness through optimized deep feature processing, thereby improving the precision of emergency vehicle localization. In Improvement 2, we propose the AIDPB module as a replacement for the original CCFM feature fusion mechanism. This modification achieves a remarkable 3.8% increase in recall (R-value). The AIDPB module excels in fusing low-level and high-level semantic information, ensuring the model can effectively focus on and recognize vehicles of varying sizes, even in complex and dynamic environments. Improvement 3, representing the final integrated enhancement of this study, delivers a 0.6% improvement in detection accuracy while simultaneously reducing floating-point operations (FLOPs) to 49.5 G and decreasing the total parameter count by 27.1%. This set of improvements demonstrates that the final model not only enhances detection accuracy but also significantly improves computational efficiency. Compared to the RT-DETR-r18 model, the proposed model exhibits substantial advantages in detection performance, making it well suited for practical emergency vehicle detection applications.

### 4.6. Effective Receptive Field (ERF)

Given that model performance is directly influenced by its receptive field size and feature extraction capability, we employ gradient visualization techniques to conduct an in-depth analysis and comparison of the effective receptive field of the optimized model [37]. In this study, we randomly select 50 images from the test set and standardize their resolution to 640 × 640 pixels. Next, we perform normalization processing on the contributions of image pixel features, ensuring that all contribution values fall within a standardized range of 0 to 1. Subsequently, we measure the effective pixel region ratio corresponding to each pixel during feature mapping. Specifically, Table 6 presents a detailed comparative analysis of the feature-effective pixel contribution rates between RT-DETR-r18 and RT-DETR-EVD under predefined thresholds of pixel contribution rates set at t = 20%, 30%, 50%, and 99%.

**Figure 10:**
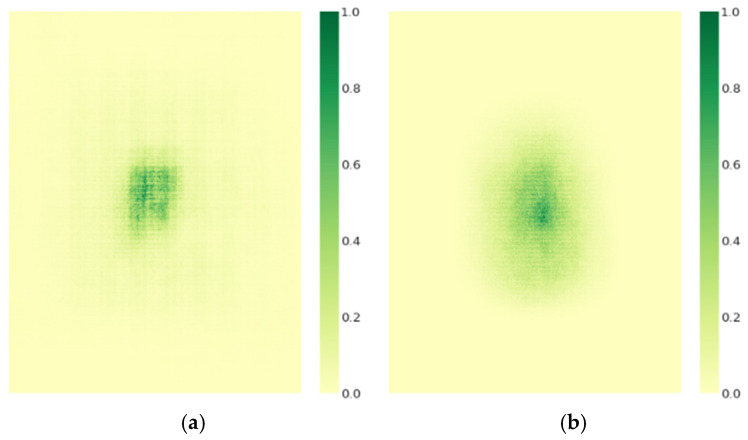
Comparison of receptive field sizes. (**a**) Receptive field distribution of RT-DETR-r18. (**b**) Receptive field distribution of RT-DETR-EVD.

As shown in Table 6, RT-DETR-EVD demonstrates a significant increase in the proportion of effective pixels compared to RT-DETR-r18 across different pixel contribution thresholds. This improvement is particularly notable at t = 99%, where the effective pixel ratio reaches 92.3%. As illustrated in Figure 10, the receptive field of RT-DETR-EVD is more uniformly distributed and effectively covers critical regions, leading to enhanced detection accuracy and greater model robustness. The comparative analysis of receptive fields indicates that RT-DETR-EVD excels in feature extraction, offering a more optimized receptive field distribution. This expansion in the effective receptive field has a significant impact on emergency vehicle detection tasks, further validating the effectiveness of the proposed improvements in this study.

### 4.7. Comparison Results of Multiple Models

**Figure 11:**
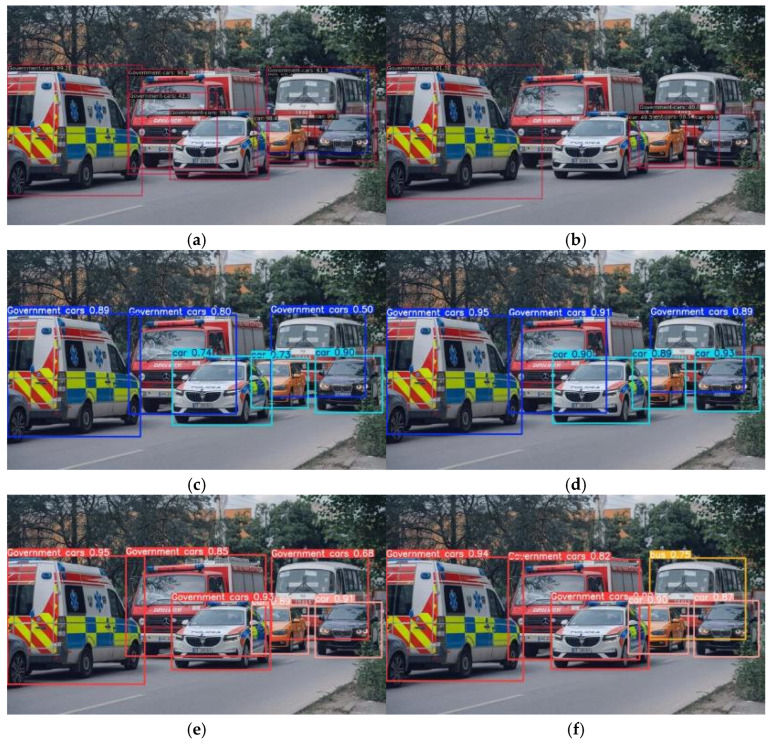
Comparison of the detection results across different models. (**a**) Faster-RCNN; (**b**) SSD; (**c**) YOLOv8m; (**d**) YOLOv11m; (**e**) RT-DETR-r18; (**f**) RT-DETR-EVD.

To comprehensively assess the performance of RT-DETR-EVD in emergency vehicle detection, we compare it against six widely used detection models: Faster-RCNN, SSD, YOLOv8m, YOLOv11m, RT-DETR-r18, and RT-DETR-EVD. All models are trained from scratch and evaluated under identical datasets and experimental conditions to ensure a fair and consistent comparison. The comparative results are presented in Table 7. It is worth noting that, without pretrained weights and given the limited diversity of government vehicle samples in the dataset, some YOLO series models failed to correctly distinguish government vehicles from ordinary cars, as shown in Figure 11. This highlights the challenge of learning fine-grained category distinctions from limited data and suggests potential future improvements through the use of pretrained features or enhanced data augmentation strategies.

The experimental results demonstrate that the optimized emergency vehicle detection model exhibits significant advantages in detection performance. As shown in Table 7, the model achieves a precision of 90.4%, a recall of 79.2%, and an mAP50 (%) of 88.3%, ranking among the top across all three key metrics. Notably, although YOLOv11n is the most lightweight model in terms of computational cost (6.5 GFLOPs), its detection accuracy is considerably lower (mAP50 of 82.4%). In comparison, the proposed model achieves a superior balance—maintaining high detection accuracy while still achieving substantial architectural optimization, with only 14.5 M parameters and 49.5 GFLOPs, both among the lowest in its category. Compared to the baseline RT-DETR-r18, the proposed model reduces the parameter count by 27.1% and the number of floating-point operations by 13.2% while also achieving a significant improvement in inference speed—reaching 54.1 FPS. This demonstrates a superior balance between detection accuracy and runtime efficiency among models of similar complexity. This dual breakthrough in accuracy and efficiency makes the proposed model highly suitable for real-time edge computing applications in emergency vehicle detection.

To systematically validate the effectiveness of the proposed improved algorithm, we conducted comparative experiments on six mainstream object detection models for emergency vehicle recognition in complex traffic scenarios. The visualized results are presented in Figure 11. Figure 10a,b illustrate the detection results of the two-stage detector Faster R-CNN and the single-stage model SSD, respectively. These results reveal significant missed detections and false positives in occluded vehicle regions, which can be attributed to the lack of cross-level feature interaction mechanisms in traditional network architectures. As a result, multi-scale information integration remains insufficient, making it difficult to capture the associations between local details and global semantics effectively. Figure 11c,e present the detection performance of YOLOv8m, YOLOv9m, and RT-DETR-r18, which incorporate progressive feature fusion strategies. Although these models partially enhance feature integration through feature pyramid structures and demonstrate improved detection of occluded objects, they still suffer from high false positive rates in complex background environments. Figure 11f showcases the detection results of the proposed RT-DETR-EVD model. By introducing a cross-scale dynamic feature interaction module and leveraging adaptive bias learning, the model constructs a multi-scale context-aware network, enabling more effective feature fusion across different scales. This significantly enhances feature discrimination in occlusion and deformation scenarios. The visualization results confirm that the proposed model exhibits superior feature differentiation and more precise object localization performance in complex traffic environments.

## 5. Discussion

The proposed RT-DETR-EVD model demonstrates promising engineering advantages, making it well suited for deployment in smart city applications. It adopts a streamlined architecture with 14.5 million parameters and 49.5 GFLOPs, striking a reasonable balance between detection accuracy and computational cost. While the parameter size may not be extremely lightweight compared to the smallest detection models, it remains efficient enough for real-time inference on edge devices, such as NVIDIA Jetson platforms and embedded CPUs integrated into roadside camera systems.

Several practical application scenarios are envisioned, including the following:

1. Emergency Traffic Signal Priority (ETSP) systems, which detect ambulances or fire trucks in real time and trigger adaptive signal control to provide unobstructed passage.
2. Roadside cameras and edge nodes, enabling distributed emergency vehicle coordination and communication with centralized traffic control centers.
3. Vehicle-to-Infrastructure (V2I) networks, broadcasting emergency vehicle detections to nearby vehicles and infrastructure for coordinated responses.

In addition, the proposed model demonstrates robust performance in occlusion and dense traffic conditions, enhancing reliability in real-world deployments. The high detection precision and recall rates support timely traffic clearance for emergency vehicles, which can help reduce response times and potentially save lives.

Nonetheless, a key limitation remains: although the model can classify emergency vehicle categories (e.g., police cars, fire trucks, ambulances) with high accuracy, it cannot directly infer whether a given vehicle is actively responding to an emergency. To address this limitation, RT-DETR-EVD can serve as a perceptual front end, integrated with complementary sources such as acoustic alarm recognition, vehicle trajectory analysis, or V2I message parsing.

In addition, while the dataset used in this study includes diverse weather conditions and complex traffic scenarios, it focuses on Russian government vehicles. This geographic and regulatory specificity may limit the model’s generalizability to other regions with different vehicle appearances, emergency markings, and traffic norms. To enhance global deployment potential, future work will involve validating the model on additional datasets featuring emergency vehicles from various countries and environments, enabling more robust cross-regional performance.

Future research will thus focus on both developing multimodal fusion frameworks and conducting cross-domain evaluations to achieve a more intelligent and adaptable emergency response system for real-world urban deployments.

## 6. Conclusions

To address the common challenges faced by traditional emergency vehicle detection methods, including insufficient accuracy, high computational cost, and poor adaptability to complex environments, this study explores a lightweight yet high-precision detection model. By introducing CSPDarknet as the new backbone network and proposing the CSP-MogaBlock hybrid architecture, the model achieves adaptive multi-level feature integration while reducing model complexity. Additionally, the incorporation of the AIDPB module, enhanced with dynamic position bias (DPB), enables the model to dynamically adjust the correlation strength between different spatial positions, effectively capturing positional dependencies within sequences and further improving feature discrimination capability. These improvements not only enhance feature extraction efficiency but also significantly reduce computational complexity, thereby optimizing the model’s overall performance and operational efficiency.

The proposed RT-DETR-EVD model demonstrates remarkable performance in emergency vehicle detection, substantiated by comprehensive experimental results. Compared to the baseline RT-DETR-r18, our model achieves significant computational efficiency improvements: parameter count reduced to 14.5 M (27% reduction) and computational complexity decreased to 49.5 G FLOPs (13.2% reduction). Performance metrics reveal noteworthy enhancements: precision increased by 0.5% and recall and mean average precision (mAP50) improved by 0.6%, with mAP50 reaching an impressive 88.3%. Notably, the model exhibits exceptional feature extraction capabilities, with effective pixel coverage reaching 92.3% at the 99% contribution threshold. These substantial improvements not only validate the proposed methodology but also underscore the RT-DETR-EVD model’s potential for high-precision emergency vehicle detection in real-world applications.

The proposed RT-DETR-EVD model demonstrates outstanding detection performance, offering a lightweight yet highly accurate solution for emergency vehicle detection. This research provides new insights and methodologies for addressing challenges in emergency vehicle detection tasks.

Although this study has achieved promising results in enhancing the detection accuracy of emergency vehicles and improving model efficiency, several limitations remain. First, the current model primarily relies on static image-based detection and has yet to incorporate temporal sequence information or multimodal inputs (e.g., acoustic signals or vehicle trajectory data), which constrains its ability to assess dynamic emergency scenarios. Second, this study focuses on classifying vehicle types but does not address whether the detected vehicles are actively engaged in emergency duties, such as operating with sirens or flashing lights. Future work will aim to extend the model to video-level detection, integrate multi-source sensing modules, explore convergence strategies for visual perception and V2X communications, and conduct field deployments in real-world urban intersections to further improve the model’s adaptability and practical utility.

## Figures and Tables

**Table 1:** Category distribution of the dataset.

Dataset		Total Images	Total Labels	Single Labels
Train	Government Car	4860	20,482	1038
	Car			16,474
	Truck			2678
	Bus			292
Val	Government Car	681	2934	116
	Car			2405
	Truck			377
	Bus			36
Test	Government Car	699	3039	105
	Car			2461
	Truck			429
	Bus			44

**Table 2:** Category distribution of the adjusted dataset.

Dataset		Total Images	Total Labels	Single Labels
Train	Government Car	4860	20,482	1038
	Car			16,474
	Truck			2678
	Bus			292
Val	Government Car	1380	5973	221
	Car			4866
	Truck			806
	Bus			80

**Table 3:** Main parameters of the model.

Training Parameter	Value
Epoch	200
Input size	640 × 640
Optimizer	AdamW
Batch size	4
Momentum	0.9
Initial learning rate	0.0001
Weight decay	0.0001
Warm-up epoch	2000

**Table 4:** Comparison of different backbone networks.

Backbone Network	Parameters/M	FLOPs/G	Mean Average Precision mAP/%
ResNet18	19.9	57.0	87.8
ResNet34	31.1	88.8	87.0
ResNet50	42.0	129.6	87.5
ResNet101	74.7	247.1	87.9
HGNetV2	32.0	103.4	87.5

**Table 5:** The results of the ablation experiments.

Model	Moga Block	AIDPB	P (%)	R (%)	mAP50 (%)	Parameters (M)	FLOPs/G
RT-DETR-r18	×	×	89.9	78.6	87.7	19.9	57.0
Improvement 1	√	×	85.3	82.3	87.7	14.7	49.7
Improvement 2	×	√	85.7	**82.4**	85.8	19.8	57.2
Improvement 3	√	√	**90.4**	79.2	**88.3**	**14.5**	**49.5**

**Table 6:** Comparison of effective receptive fields.

	**t = 20%**	**t = 30%**	**t = 50%**	**t = 99%**
RT-DETR-r18	1.7%	2.8%	5.7%	29.4%
RT-DETR-EVD	1.8%	2.9%	8.4%	92.3%

**Table 7:** Performance comparison of different models.

Model	P (%)	R (%)	mAP50 (%)	mAP95 (%)	Parameters (M)	FLOPs (G)	FPS (f/s)
Faster-RCNN	83.8	75.9	84.9	67.5	41.4	194	27.6
SSD	73.8	63.2	68.3	45.9	24,1	315	26.5
YOLOv8m	89.3	79.1	87.8	**74.5**	25.8	78.7	**146.6**
YOLOv11m	85.6	79.2	86.4	73.1	20.1	67.7	113.7
YOLOv11n	82.8	75.7	82.4	66.5	2.6	6.3	133.2
RT-DETR-r18	89.9	78.6	87.7	72.8	19.9	57.0	50.7
Ours	**90.4**	**79.2**	**88.3**	72.4	**14.5**	**49.5**	54.1

## Data Availability

The data that support the findings of this study are openly available in Kaggle at https://www.kaggle.com/datasets/k1rsn7/recognition-of-government-vehicles-in-russia (accessed on 5 July 2024).
